# Research of the lipoprotein combined index and its association with osteoporosis in elderly Chinese adults: a retrospective cross-sectional study

**DOI:** 10.7717/peerj.21518

**Published:** 2026-07-14

**Authors:** Ming Xu, Meiling Zhu, Lixiang Zhang, Lingyun Tian, Xinmin Chu

**Affiliations:** 1 Department of Health Management Center, The First Affiliated Hospital of USTC, Division of Life Science and Medicine, University of Science and Technology of China, Hefei, Anhui, China; 2 Department of Cardiology, The First Affiliated Hospital of USTC, Division of Life Science and Medicine, University of Science and Technology of China, Hefei, Anhui, China

**Keywords:** Quantitative CT, Elderly adults, Osteoporosis, Lipoprotein combined index

## Abstract

**Objective:**

This study aimed to investigate the relationship between the lipoprotein combined index (LCI) and osteoporosis (OP) risk in elderly Chinese individuals.

**Methods:**

This retrospective cross-sectional study included 7,383 participants aged 60 years and older who underwent chest computed tomography (CT) with quantitative CT (QCT) at the First Affiliated Hospital of the University of Science and Technology of China, Hefei, from January 2023 to June 2025. Participants were categorized into non-osteoporosis (non-OP) and osteoporosis (OP) groups based on lumbar spine bone mineral density (BMD), with BMD thresholds set at ≥ 80 mg/cm^3^ for non-OP and < 80 mg/cm^3^ for OP. The LCI was calculated using the following formula: (total cholesterol × triglycerides × low-density lipoprotein cholesterol (LDL-C))/high-density lipoprotein cholesterol (HDL-C), and was divided into quartiles (Q1–Q4). Multivariate logistic regression, restricted cubic spline (RCS) plots, and subgroup analyses were used to assess the relationship between LCI and OP.

**Results:**

LCI levels were significantly lower in the OP group compared to the non-OP group (*P* < 0.001). After multivariate adjustment, higher LCI levels were inversely associated with OP risk (OR = 0.993, 95% CI [0.990–0.997], *P* < 0.001). Specifically, individuals in the Q4 group had a 19.3% lower risk of OP than those in the Q1 group (OR = 0.807, 95% CI [0.681–0.956], *P* = 0.013). RCS analysis revealed a nonlinear relationship (*P* = 0.048), with a threshold effect at an LCI of 18; below this threshold, no significant correlation was found, whereas above it, each unit increase in LCI was associated with a 1.0% reduction in risk. Subgroup analysis indicated that the inverse association of LCI was more pronounced in participants without a history of cerebral infarction (*P* for interaction = 0.003).

**Conclusion:**

Higher LCI levels are independently linked to a reduced risk of OP in older adults, particularly when LCI ≥ 18. LCI may serve as a potential biomarker reflecting the role of lipid metabolism in OP.

## Introduction

Osteoporosis is a systemic skeletal disorder characterized by reduced bone mass and microarchitectural deterioration, leading to weakened bone strength and an increased risk of fractures (Johnston & Dagar, 2020). With the aging global population, osteoporosis has emerged as a major public health issue in the 21st century. Fragility fractures represent the most severe clinical consequence of the condition (Working Group of the 2023 Chinese Guidelines for the Diagnosis and Treatment of Senile Osteoporosis et al., 2023). Epidemiological studies indicate that approximately 33% of women and 20% of men aged 50 years and older will experience osteoporotic fractures (Liu et al., 2024). These fractures severely affect patients’ quality of life and functional capacity while imposing significant economic burdens on healthcare systems (Zhang et al., 2025).

Recent research highlights a complex interaction between lipoproteins and osteoporosis, involving lipid metabolism, bone homeostasis, and inflammatory processes (Zhang et al., 2024; Anagnostis et al., 2022). However, identifying reliable lipid-based predictors of bone mineral density (BMD) remains challenging, with inconsistent evidence regarding specific lipoproteins (Sun et al., 2024; Tang et al., 2021; Zolfaroli et al., 2021). Some studies report positive correlations between high-density lipoprotein cholesterol (HDL-C) and BMD (Zolfaroli et al., 2021; Xie et al., 2022), while others find negative or no significant associations (Tang et al., 2021). Similarly, low-density lipoprotein cholesterol (LDL-C) has been linked to decreased BMD in individuals with osteoporosis (Cui et al., 2024), although some studies fail to establish a significant relationship (Zhao et al., 2023). Even composite markers such as non-HDL-C and the non-HDL-C/HDL-C ratio show inconsistent, sometimes L-shaped, associations with low bone mass (Serés-Noriega et al., 2023; Chen et al., 2025; Qi et al., 2024; Wang et al., 2024). This inconsistency highlights the need for a more comprehensive lipid biomarker capable of integrating multiple metabolic parameters to assess bone health.

The Lipoprotein Combined Index (LCI) has been proposed as a potential integrated biomarker. By combining total cholesterol (TC), triglycerides (TG), LDL-C, and HDL-C (Qiu et al., 2025; Si et al., 2020), the LCI provides a holistic lipid profile that may address the limitations of individual lipid parameters in predicting bone health outcomes. While the LCI has proven valuable in other metabolic and vascular contexts—such as assessing risks for cardiovascular disease (CVD) (Qiu et al., 2024; Arsenault, Boekholdt & Kastelein, 2011), predicting adverse outcomes in myocardial infarction (Drwila et al., 2022), and evaluating coronary artery disease (CAD) (Mahdavi-Roshan et al., 2022; Yilmaz et al., 2025) or atherosclerosis in diabetic patients (Serés-Noriega et al., 2023)—its role in bone health remains underexplored. Previous studies suggest its superior predictive value in vascular health (Mahdavi-Roshan et al., 2022; Yilmaz et al., 2025), but research on the relationship between LCI and osteoporosis is limited, often constrained by small sample sizes. Large-scale studies are necessary to determine whether the LCI can serve as a reliable biomarker for osteoporosis. This study employs quantitative computed tomography (QCT) to assess BMD in an elderly cohort, aiming to clarify the association between LCI and osteoporosis and inform early prevention strategies.

## Objects and methods

### Research objects

This retrospective cross-sectional study included 7,383 elderly individuals (aged ≥ 60 years) who underwent low-dose chest computed tomography (CT) along with QCT for BMD assessment at the Department of Health Management Center, First Affiliated Hospital of the University of Science and Technology of China, Hefei, from January 2023 to June 2025.

Inclusion criteria were as follows: (1) participants aged 60 years or older; (2) completion of both low-dose chest CT and QCT BMD analysis; and (3) routine fasting blood tests. Exclusion criteria included: (1) incomplete or extreme clinical data; (2) QCT images failing to meet required standards or with incomplete or extreme measurement indices; (3) liver or renal insufficiency, thyroid disorders, malignancies, severe organic diseases, or other significant medical conditions; (4) recent acute or chronic infections; and (5) a history of lumbar fracture or surgery. The study protocol was approved by the Institutional Review Board of the First Affiliated Hospital of the University of Science and Technology of China (No. 2025-RE-243; dated September 22, 2025) and conducted in accordance with the Declaration of Helsinki. Given the retrospective design, the ethics committee waived the informed consent requirement.

### Data collection and measurements

This retrospective cross-sectional study employed de-identified patient data extracted from the hospital’s health management system. The dataset was accessed by the research team on September 24, 2025, solely for analysis purposes. The data included the following categories: (1) Demographic information, such as sex, age, and marital status; (2) medical history, including conditions like hypertension, diabetes mellitus, coronary heart disease, cerebral infarction, atrial fibrillation, and hyperuricemia; (3) physical examination data, which consisted of measurements of pulse, systolic and diastolic blood pressure, as well as height and weight. Weight and height were used to calculate body mass index (BMI) using the formula: BMI = weight (kg)/height (m)^2^; (4) laboratory test results from fasting venous blood samples, which were analyzed for biochemical indicators (*e.g.*, albumin, blood urea nitrogen, creatinine, uric acid), complete blood count parameters (*e.g.*, red blood cell count, platelet count, white blood cell count, hemoglobin levels), and lipoprotein profiles, including TG, TC, LDL-C, and HDL-C. These lipid parameters were used to calculate the LCI using the formula: LCI = TC × TG × LDL-C/HDL-C (Qiu et al., 2025; Si et al., 2020).

Lumbar BMD Measurement Protocol: All participants underwent standardized low-dose chest CT scans using a 64-row GE Lightspeed VCT scanner, with the scanning range extending to the inferior margin of the L2 vertebra (Cheng et al., 2020). Prior to scanning, the equipment was calibrated with the European Spine Phantom (No. 145). The scanning parameters included a tube voltage of 120 kV, a tube current of 100 mA, a field of view of 500× 500 mm, a slice thickness of five mm, and a pitch of 0.984. The acquired images were transferred to the QCT Pro workstation (Version 6.1), where certified radiologists performed post-processing analyses. Using the BMD measurement module, BMD was evaluated in the L1 and L2 vertebral bodies, excluding areas with sclerosis or bone islands. The final lumbar BMD measurement was recorded as the mean BMD value from L1 and L2 (Cheng et al., 2020). Participants were categorized into non-osteoporotic (non-OP) and osteoporotic (OP) groups based on diagnostic thresholds, with non-OP defined as BMD ≥ 80 mg/cm^3^ and OP defined as BMD <80 mg/cm^3^ (Cheng et al., 2020).

### Statistical methods

All statistical analyses were performed using R software (R Foundation for Statistical Computing, Vienna, Austria; available at http://www.R-project.org) and Empower Stats (X&Y Solutions, Boston, USA; accessible at http://www.empowerstats.com). Missing data were addressed through a complete-case analysis, excluding participants with any missing or incomplete clinical data, laboratory results, or QCT measurements during the initial screening phase. This ensured that the final dataset (*n* = 7,383) contained no missing values for the variables included in the models. Continuous data with a normal distribution are presented as mean ± standard deviation (SD) and were compared using analysis of variance (ANOVA). Categorical data are reported as frequencies and percentages (n [%]), with intergroup comparisons conducted using the chi-square test or Fisher’s exact test, as appropriate.

To examine the relationship between LCI and osteoporosis risk, three logistic regression models were applied: a crude model (Model 1, unadjusted); a minimally adjusted model (Model 2, controlling for age, sex, and marital status); and a fully adjusted model (Model 3, further adjusting for BMI, blood pressure, blood biochemical markers, and comorbidities). Odds ratios (ORs) and 95% confidence intervals (CIs) were calculated for each model. In the main analysis, LCI was treated as a continuous variable to estimate the OR per unit increase. To assess the robustness of the results and explore potential dose–response relationships, a sensitivity analysis stratified LCI into quartiles (Q1–Q4). The potential nonlinear association between LCI and osteoporosis risk was examined using a restricted cubic spline (RCS) model with three knots at the 10th, 50th, and 90th percentiles of the LCI distribution. These knot locations were selected based on standard statistical guidelines to balance model flexibility and interpretability, enabling the detection of nonlinear trends while preventing overfitting. When nonlinearity was identified (likelihood ratio test *P* < 0.05), a two-piecewise linear regression model was used to identify the inflection point (threshold). This threshold value was determined through a grid search and recursive algorithms, selecting the LCI value that maximized the log-likelihood of the two-piecewise model. ORs were subsequently calculated for each segment.

Subgroup analyses were conducted based on categorical variables, including gender and baseline comorbidities such as hypertension and diabetes. Stratified logistic regression models were used to estimate ORs for LCI within each subgroup. Interaction effects were evaluated using likelihood ratio tests, with a *P*-value of less than 0.05 for the interaction term indicating potential effect modification. Statistical significance was set at a two-sided *P*-value of less than 0.05.

## Results

### Baseline characteristic analysis

This study included 7,383 participants, who were divided into four quartiles (Q1–Q4) based on their LCI levels: Q1 (LCI < 8.69, *n* = 1,846); Q2 (LCI [8.69–15.58], *n* = 1,845); Q3 (LCI [15.59–27.42], *n* = 1,846); and Q4 (LCI > 27.42, *n* = 1,846). Significant differences in baseline characteristics were observed among the four groups (*P* < 0.05) for the following variables: age, BMI, pulse, systolic and diastolic blood pressure, LCI, albumin, urea nitrogen, uric acid, red blood cell count, platelet count, white blood cell count, hemoglobin concentration, sex, diabetes, coronary heart disease, cerebral infarction, atrial fibrillation, and osteoporosis. No significant differences were found for creatinine, marital status, hypertension, or hyperuricemia (Table 1).

**Table 1:** Baseline characteristic analysis.

Variables	Total (*n* = 7383)	Q1 (<8.69, *n* = 1846)	Q2 (8.69–15.58, *n* = 1845)	Q3 (15.59–27.42, *n* = 1846)	Q4 (>27.42, *n* = 1846)	Statistic	*P*
Age, Mean ± SD	68.43 ± 7.87	70.00 ± 8.48	68.85 ± 8.02	68.03 ± 7.63	66.85 ± 6.92	*F* = 53.71	**<0.001**
Body mass index, Mean ± SD	24.20 ± 2.99	23.32 ± 3.08	23.97 ± 3.02	24.44 ± 2.80	25.06 ± 2.80	*F* = 117.98	**<0.001**
Pulse, Mean ± SD	78.85 ± 11.95	77.62 ± 11.87	78.28 ± 11.60	79.14 ± 12.27	80.33 ± 11.90	*F* = 17.89	**<0.001**
Systolic blood pressure, Mean ± SD	135.48 ± 18.45	133.71 ± 18.58	135.09 ± 18.67	136.25 ± 18.15	136.88 ± 18.26	*F* = 10.57	**<0.001**
Diastolic pressure, Mean ± SD	77.42 ± 10.74	75.17 ± 10.53	76.78 ± 10.95	78.21 ± 10.40	79.50 ± 10.60	*F* = 56.95	**<0.001**
Lipoprotein combined index, Mean ± SD	21.29 ± 27.38	5.46 ± 2.00	11.94 ± 1.99	20.93 ± 3.39	46.85 ± 44.58	*F* = 1215.69	**<0.001**
Serum albumin, Mean ± SD	44.30 ± 2.25	43.92 ± 2.40	44.15 ± 2.16	44.35 ± 2.17	44.76 ± 2.16	*F* = 47.11	**<0.001**
Blood urea nitrogen, Mean ± SD	6.02 ± 1.62	6.11 ± 1.62	6.04 ± 1.61	6.00 ± 1.57	5.94 ± 1.67	*F* = 3.55	**0.014**
Serum creatinine, Mean ± SD	71.01 ± 23.04	70.43 ± 24.62	70.52 ± 22.16	70.94 ± 18.57	72.13 ± 26.10	*F* = 2.14	0.093
Serum uric acid, Mean ± SD	341.90 ± 81.03	320.08 ± 76.32	332.44 ± 78.46	348.90 ± 79.14	366.16 ± 82.63	*F* = 118.08	**<0.001**
Red blood cell count, Mean ± SD	4.63 ± 0.45	4.51 ± 0.46	4.57 ± 0.43	4.68 ± 0.43	4.77 ± 0.43	*F* = 130.13	**<0.001**
Platelet count, Mean ± SD	210.22 ± 54.02	197.47 ± 53.64	207.73 ± 51.50	213.46 ± 53.28	222.20 ± 54.66	*F* = 69.95	**<0.001**
White blood cell count, Mean ± SD	6.08 ± 1.57	5.75 ± 1.53	5.97 ± 1.56	6.17 ± 1.53	6.43 ± 1.56	*F* = 63.64	**<0.001**
Hemoglobin concentration, Mean ± SD	141.60 ± 13.01	138.30 ± 13.29	139.86 ± 12.44	142.80 ± 12.51	145.45 ± 12.61	*F* = 114.77	**<0.001**
Gender, n(%)						*χ*^2^= 11.66	**0.009**
Male	4,662 (63.15)	1,195 (64.73)	1,105 (59.89)	1,175 (63.65)	1,187 (64.30)		
Female	2,721 (36.85)	651 (35.27)	740 (40.11)	671 (36.35)	659 (35.70)		
Marital status, n(%)						–	0.474
Unmarried	14 (0.19)	4 (0.22)	1 (0.05)	5 (0.27)	4 (0.22)		
Married	7,369 (99.81)	1,842 (99.78)	1,844 (99.95)	1,841 (99.73)	1,842 (99.78)		
Hypertension, n(%)						*χ*^2^= 1.16	0.762
No	5,002 (67.75)	1,232 (66.74)	1,258 (68.18)	1,256 (68.04)	1,256 (68.04)		
Yes	2,381 (32.25)	614 (33.26)	587 (31.82)	590 (31.96)	590 (31.96)		
Diabetes, n(%)						*χ*^2^= 47.56	**<0.001**
No	6,588 (89.23)	1,573 (85.21)	1,652 (89.54)	1,665 (90.20)	1,698 (91.98)		
Yes	795 (10.77)	273 (14.79)	193 (10.46)	181 (9.80)	148 (8.02)		
Coronary heart disease, n(%)						*χ*^2^= 134.87	**<0.001**
No	7,112 (96.33)	1,705 (92.36)	1,770 (95.93)	1,810 (98.05)	1,827 (98.97)		
Yes	271 (3.67)	141 (7.64)	75 (4.07)	36 (1.95)	19 (1.03)		
Cerebral infarction, n(%)						*χ*^2^= 25.56	**<0.001**
No	7,306 (98.96)	1,816 (98.37)	1,815 (98.37)	1,834 (99.35)	1,841 (99.73)		
Yes	77 (1.04)	30 (1.63)	30 (1.63)	12 (0.65)	5 (0.27)		
Atrial fibrillation, n(%)						*χ*^2^= 25.48	**<0.001**
No	7,332 (99.31)	1,818 (98.48)	1,835 (99.46)	1,838 (99.57)	1,841 (99.73)		
Yes	51 (0.69)	28 (1.52)	10 (0.54)	8 (0.43)	5 (0.27)		
Hyperuricemia, n(%)						*χ*^2^= 5.92	0.115
No	7,281 (98.62)	1,829 (99.08)	1,821 (98.70)	1,819 (98.54)	1,812 (98.16)		
Yes	102 (1.38)	17 (0.92)	24 (1.30)	27 (1.46)	34 (1.84)		
Osteoporosis, n(%)						*χ*^2^= 37.51	**<0.001**
No	5,437 (73.64)	1,297 (70.26)	1,317 (71.38)	1,377 (74.59)	1,446 (78.33)		
Yes	1,946 (26.36)	549 (29.74)	528 (28.62)	469 (25.41)	400 (21.67)		

**Notes.**

F ANOVA *χ*^2^ Chi-square test - Fisher exact SD standard deviation; Bold values indicate *P* < 0.05

### Results of the analysis on the association between LCI and osteoporosis

A multivariate logistic regression model was employed to assess the relationship between LCI and osteoporosis risk. The results, including both unadjusted and adjusted models, are presented in Table 2. In the unadjusted model (Model 1), each one-unit increase in LCI (treated as a continuous variable) was associated with a 1.2% reduction in the risk of osteoporosis (OR = 0.988, 95% CI [0.984–0.991], *P* < 0.001). After minimal adjustments for sex, marital status, and age (Model 2), this association remained statistically significant, though slightly attenuated (OR = 0.992, 95% CI [0.988–0.995], *P* < 0.001). Additional adjustments for 17 covariates, including BMI, comorbidities, and laboratory parameters (Model 3), continued to support this inverse association (OR = 0.993, 95% CI [0.990–0.997], *P* < 0.001).

**Table 2:** Results of the analysis on the association between LCI and osteoporosis.

Variables	Model1		Model2		Model3	
	OR (95% CI)	*P*	OR (95% CI)	*P*	OR (95% CI)	*P*
LCI	0.988 (0.984∼0.991)	**<0.001**	0.992 (0.988∼0.995)	**<0.001**	0.993 (0.990∼0.997)	**<0.001**
LCI is grouped by quartile						
Q1	1.000 (Reference)		1.000 (Reference)		1.000 (Reference)	
Q2	0.947 (0.822∼1.092)	0.453	0.983 (0.842∼1.148)	0.827	0.972 (0.836∼1.131)	0.715
Q3	0.805 (0.696∼0.930)	**0.003**	0.922 (0.787∼1.080)	0.316	0.917 (0.782∼1.074)	0.282
Q4	0.654 (0.563∼0.759)	**<0.001**	0.823 (0.699∼0.969)	**0.019**	0.807 (0.681∼0.956)	**0.013**
P for trend		**<0.001**		**0.015**		**0.012**

**Notes.**

OR Odds Ratio CI Confidence Interval Model1 Crude Model2 Adjust: gender, marital status, age Model3 Adjust: gender, marital status, hypertension, diabetes, coronary heart disease, cerebral infarction, atrial fibrillation, hyperuricemia, age, body mass index, pulse, systolic blood pressure, diastolic blood pressure, albumin, urea nitrogen, creatinine, uric acid, red blood cell count, platelet count, white blood cells and hemoglobin concentration; Bold values indicate *P* < 0.05

To validate the main analysis, a sensitivity analysis was conducted by stratifying LCI into quartiles (Q1–Q4). A significant inverse dose–response relationship was observed between LCI levels and osteoporosis risk, with trend *P*-values of <0.001 in Model 1 and 0.012 in Model 3. In Model 3, fully adjusted for all covariates, individuals in the highest LCI quartile (Q4) had a 19.3% lower risk of osteoporosis compared to those in the lowest quartile (Q1) (OR = 0.807, 95% CI [0.681–0.956], *P* = 0.013). No significant differences were found for the intermediate quartiles (Q2–Q3; all *P* > 0.05). These findings further reinforce the inverse relationship between LCI and osteoporosis risk. Detailed results are presented in Table 3.

**Table 3:** Threshold effect analysis of LCI on osteoporosis.

Regression model	OR (95% CI)^*^	*P*-value
Fitting by standard Logistic regression model	0.993 (0.990, 0.997)	<0.001
Fitting by piecewise Logistic regression model (break-point = 18)		
Lipoprotein combined index <18	1.005 (0.993, 1.018)	0.407
Lipoprotein combined index ≥ 18	0.990 (0.985, 0.995)	<0.001
Log likelihood ratio		0.048

**Notes.**

* Adjusted for: gender, age, marital status, hypertension, diabetes, coronary heart disease, cerebral infarction, atrial fibrillation, hyperuricemia, body mass index, pulse, systolic blood pressure, diastolic blood pressure, albumin, urea nitrogen, creatinine, uric acid, red blood cell count, platelet count, white blood cells and hemoglobin concentration.

### Analysis of the threshold effect of LCI on osteoporosis

LCI, as a continuous variable, was examined for its potential nonlinear relationship with osteoporosis using an RCS model. The analysis adjusted for various covariates, including demographic factors (sex, age, marital status), comorbidities (hypertension, diabetes, coronary heart disease, cerebral infarction, atrial fibrillation, hyperuricemia), anthropometric measures (BMI, pulse rate, systolic and diastolic blood pressure), and laboratory parameters (albumin, urea nitrogen, creatinine, uric acid, red blood cell count, platelet count, white blood cell count, and hemoglobin concentration).

**Figure 1:**
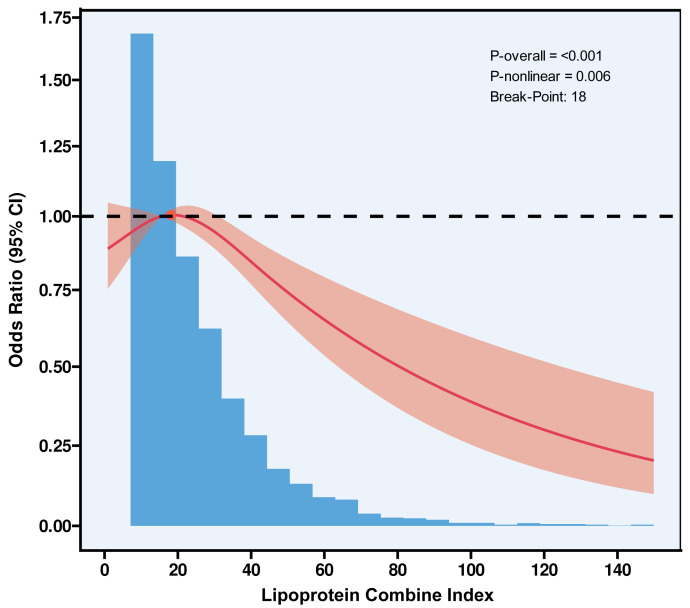
RCS curve of the association between LCI and osteoporosis. A nonlinear relationship between them was detected after adjusting for gender, age, marital status, hypertension, diabetes, coronary heart disease, cerebral infarction, atrial fibrillation, hyperuricemia, body mass index, pulse, systolic blood pressure, diastolic blood pressure, albumin, urea nitrogen, creatinine, uric acid, red blood cell count, platelet count, white blood cells and hemoglobin concentration.

A nonlinear association was identified (*P* = 0.048, likelihood ratio test), with an inflection point at an LCI of 18 (refer to Fig. 1). For LCI values below this threshold (LCI < 18), no significant association with osteoporosis risk was observed (OR = 1.005, 95% CI [0.993–1.018], *P* = 0.407). In contrast, for LCI values ≥ 18, each unit increase in LCI was linked to a 1.0% reduction in osteoporosis risk (OR = 0.990, 95% CI [0.985–0.995], *P* < 0.001). These results suggest a dose-dependent inverse association of higher LCI levels beyond this threshold. A full presentation of the results is provided in Table 3.

### Subgroup analysis of the association between LCI and osteoporosis

Subgroup analyses were performed to explore potential effect modification by baseline characteristics (Table 4). A significant interaction was observed only within the cerebral infarction subgroup (P for interaction = 0.003), while no significant interactions were found for sex (*P* = 0.707), marital status (*P* = 0.783), hypertension (*P* = 0.654), diabetes (*P* = 0.723), or coronary heart disease (*P* = 0.566). Notably, the relationship between LCI and osteoporosis differed substantially based on cerebral infarction status. Among participants without cerebral infarction, each unit increase in LCI was associated with a 1% reduction in the risk of osteoporosis (OR = 0.99, 95% CI [0.99–1.00], *P* < 0.001). In contrast, among those with cerebral infarction, each unit increase in LCI was linked to a 13% increase in the risk of osteoporosis (OR = 1.13, 95% CI [1.02–1.26], *P* = 0.022). All models were adjusted for 21 potential confounders, including sex, age, and comorbidities, but the stratification variables themselves were not included in the adjustment sets.

**Table 4:** Subgroup analysis of the association between LCI and osteoporosis.

Subgroup	n (%)	OR (95%CI)	*P-value*	P for interaction
All patients	7383 (100.00)	0.99 (0.99∼1.00)	**<0.001**	
Gender				0.707
Male	4662 (63.15)	0.99 (0.99∼1.00)	**0.012**	
Female	2721 (36.85)	0.99 (0.99∼1.00)	**0.012**	
Marital status				0.783
Unmarried	14 (0.19)	0.48 (0.00∼Inf)	1.000	
Married	7,369 (99.81)	0.99 (0.99∼1.00)	**<0.001**	
Hypertension				0.654
No	5,002 (67.75)	1.00 (0.99∼1.00)	**0.033**	
Yes	2,381 (32.25)	0.99 (0.98∼1.00)	**<0.001**	
Diabetes				0.723
No	6,588 (89.23)	0.99 (0.99∼1.00)	**0.001**	
Yes	795 (10.77)	0.99 (0.97∼1.00)	**0.05**	
Coronary heart disease				0.569
No	7,112 (96.33)	0.99 (0.99∼1.00)	**<0.001**	
Yes	271 (3.67)	0.98 (0.96∼1.01)	0.180	
Cerebral Infarction				**0.003**
No	7,306 (98.96)	0.99 (0.99∼1.00)	**<0.001**	
Yes	77 (1.04)	1.13 (1.02∼1.26)	**0.022**	
Atrial fibrillation				0.47
No	7,332 (99.31)	0.99 (0.99∼1.00)	**<0.001**	
Yes	51 (0.69)	0.96 (0.87∼1.07)	0.511	
Hyperuricemia				0.544
No	7,281 (98.62)	0.99 (0.99∼1.00)	**<0.001**	
Yes	102 (1.38)	2.85 (0.00∼Inf)	1.000	

**Notes.**

Note 1: Above model adjusted for gender, age, marital status, hypertension, diabetes, coronary heart disease, cerebral infarction, atrial fibrillation, hyperuricemia, body mass index, pulse, systolic blood pressure, diastolic blood pressure, albumin, urea nitrogen, creatinine, uric acid, red blood cell count, platelet count, white blood cells and hemoglobin concentration.

Note 2:In each case, the model is not adjusted for the stratification variable; Bold values indicate *P* < 0.05.

## Discussion

This study investigated the relationship between the LCI and osteoporosis risk in the elderly population through a cross-sectional analysis of a large sample. The results show that higher LCI levels are independently associated with a reduced risk of osteoporosis, with a notably stronger inverse association when LCI ≥ 18. These findings provide new clinical evidence on the interplay between lipid metabolism and bone health.

The LCI, a composite index that integrates TC, TG, LDL-C, and HDL-C, offers a more comprehensive assessment of lipid metabolism status. Previous studies have primarily focused on individual lipoprotein components and their relationship with BMD, often yielding inconsistent results (Anagnostis et al., 2022; Sun et al., 2024; Tang et al., 2021; Zolfaroli et al., 2021). For example, some studies have reported a positive correlation between HDL-C and BMD, suggesting that higher HDL-C levels are associated with increased BMD (Zolfaroli et al., 2021; Xie et al., 2022), while others have found no significant or negative correlations (Tang et al., 2021). Similarly, elevated LDL-C levels have been linked to reduced BMD in patients with osteoporosis (Cui et al., 2024), although other studies have not found such associations (Zhao et al., 2023).

In contrast, this study demonstrates that the LCI, which reflects the overall balance of lipid metabolism, shows a more robust association with osteoporosis risk than individual lipid components. This finding supports the hypothesis of multifactorial interactions between lipid metabolism and bone signaling pathways or diseases (Su, Li & Liao, 2019; Bai, Zhao & Hong, 2018). The threshold effect identified through RCS analysis, with a critical value of 18, suggests a potential biological inflection point: below an LCI of 18, lipid metabolic dysregulation may not significantly impact bone metabolism; however, above this threshold, lipoproteins appear to have synergistic inverse associations. This observation aligns with previous research on non-HDL-C/HDL-C ratios, which demonstrate an L-shaped correlation with low bone mass conditions, such as osteoporosis and osteopenia (Serés-Noriega et al., 2023; Chen et al., 2025; Qi et al., 2024; Wang et al., 2024), further indicating nonlinear relationships between composite lipid indices and osteoporosis.

Mechanistically, the inverse associations of LCI may be mediated through several pathways. An optimized lipoprotein ratio could reduce vascular endothelial inflammation and improve bone marrow microcirculation, thereby maintaining a physiological microenvironment that promotes bone formation (Zhi et al., 2025). Additionally, metabolic homeostasis indicated by LCI may influence bone metabolism through the regulation of adiponectin secretion (Fang et al., 2025; Huang et al., 2025; Xue et al., 2025). However, subgroup analysis revealed a positive correlation between LCI and osteoporosis in patients with cerebral infarction (*P* for interaction = 0.003). This reversal of association, where elevated LCI levels shifted from an inverse association in the general population (OR = 0.99) to a positive association in the cerebral infarction group (OR = 1.13), requires a more nuanced interpretation.

This phenomenon may be attributed to the disruption of the ”brain-bone axis” following ischemic injury. As summarized by Shi & Chen (2024), the central nervous system (CNS) regulates skeletal metabolism through neural pathways, particularly the sympathetic nervous system (SNS). In patients with prior cerebral injury, chronic SNS activation induces the release of norepinephrine, which binds to β2-adrenergic receptors (β2-ARs) on osteoblasts, leading to upregulation of RANKL expression and stimulating osteoclast-mediated bone resorption. Additionally, elevated LCI levels in this population may reflect a pro-inflammatory lipid state. Fan et al. (2025) reported that blood lipid markers and proinflammatory cytokines, such as interleukin-6 (IL-6), can synergistically exacerbate bone mass loss. In this ”vicious cycle”, high lipid levels promote IL-6 secretion, which in turn intensifies lipid metabolism disorders and accelerates the progression of osteoporosis. Therefore, neuroendocrine changes following cerebral infarction, such as activation of the hypothalamus-pituitary axis, may modify lipoprotein metabolism pathways (Zheng & Li, 2017; Chen, Zhang & Cao, 2015), negating the typically inverse associations of lipoproteins and instead advancing vascular pathology.

This study offers three key innovations compared to previous research: First, the novel application of the LCI in assessing osteoporotic risk enhances its clinical relevance. Second, the use of QCT technology for BMD measurement minimizes interference from aortic calcification, a limitation commonly associated with traditional Dual-energy X-ray Absorptiometry (DXA) (Miao et al., 2022; Li et al., 2012). Third, the large sample size (*n* = 7,383) and multivariate adjustment for 21 confounding variables significantly strengthen the robustness of the findings. Notably, Zhang et al. (2025) identified a positive correlation between the Neutrophil-to-Lymphocyte Ratio (NLR) and lumbar BMD, which contrasts with other studies, suggesting that the relationship between inflammatory markers and bone metabolism may vary depending on population characteristics and detection methods. Although this study did not directly analyze inflammatory indices, the anti-inflammatory properties of HDL-C, a key component of the LCI, suggest a potential connection. Future research could explore these underlying mechanisms by incorporating inflammatory markers such as C-reactive protein (CRP) and IL-6.

## Limitations

This study has several limitations. First, the cross-sectional design limits the ability to establish causal relationships between LCI and osteoporosis, emphasizing the need for validation through prospective cohort studies. Second, the potential impact of unmeasured confounders, such as lifestyle factors (*e.g.*, physical activity, dietary fat intake) and medication use (*e.g.*, statins), may affect the results. Moreover, the LCI calculation does not account for variations in lipoprotein subtypes (*e.g.*, HDL2 and HDL3), which could obscure deeper biological insights. Future research should aim to extend follow-up duration, improve the analysis of lipoprotein components, and conduct mechanistic studies to further validate these findings.

## Conclusion

In the elderly population, higher LCI levels are independently associated with a reduced risk of osteoporosis, with a particularly strong inverse association observed at LCI values ≥ 18. These findings suggest that LCI could serve as a comprehensive biomarker for evaluating the role of lipid metabolism in bone health, offering potential for early osteoporosis screening in older adults. However, the contrasting associations found in specific subgroups, such as those with cerebral infarction, warrant further mechanistic investigation.

## Supplemental Information

10.7717/peerj.21518/supp-1 Supplemental Information 1 The raw data The figures and tables in this manuscript are all based on the analysis of the raw data.

10.7717/peerj.21518/supp-2 Supplemental Information 2 STROBE Statement

## Abbreviations

LCI: lipoprotein combined index
OP: osteoporosis
RCS: restricted cubic spline
BMD: bone mineral density
TG: triglycerides
TC: total cholesterol
LDL-C: low-density lipoprotein cholesterol
HDL-C: high-density lipoprotein cholesterol

## Additional Information and Declarations

### Data Availability

The following information was supplied regarding data availability: The raw data is available in the Supplemental Files.
